# Mortality trends among Alaska Native people: successes and challenges

**DOI:** 10.3402/ijch.v72i0.21185

**Published:** 2013-08-05

**Authors:** Peter Holck, Gretchen Ehrsam Day, Ellen Provost

**Affiliations:** Division of Community Health Services, Alaska Native Epidemiology Center, Alaska Native Tribal Health Consortium, Anchorage, AK, USA

**Keywords:** Native American, unintentional injury, suicide, death, vital statistics

## Abstract

**Background:**

Current mortality rates are essential for monitoring, understanding and developing policy for a population's health. Disease-specific Alaska Native mortality rates have been undergoing change.

**Objective:**

This article reports recent mortality data (2004–2008) for Alaska Native/American Indian (AN/AI) people, comparing mortality rates to US white rates and examines changes in mortality patterns since 1980.

**Design:**

We used death record data from the state of Alaska, Department of Vital Statistics and SEER*Stat software from the National Cancer Institute to calculate age-adjusted mortality rates.

**Results:**

Annual age-adjusted mortality from all-causes for AN/AI persons during the period 2004–2008 was 33% higher than the rate for US whites (RR=1.33, 95% CI 1.29–1.38). Mortality rates were higher among AN/AI males than AN/AI females (1212/100,000 vs. 886/100,000). Cancer remained the leading cause of death among AN/AI people, as it has in recent previous periods, with an age-adjusted rate of 226/100,000, yielding a rate ratio (RR) of 1.24 compared to US whites (95% CI 1.14–1.33). Statistically significant higher mortality compared to US white mortality rates was observed for nine of the ten leading causes of AN/AI mortality (cancer, unintentional injury, suicide, alcohol abuse, chronic obstructive pulmonary disease [COPD], cerebrovascular disease, chronic liver disease, pneumonia/influenza, homicide). Mortality rates were significantly lower among AN/AI people compared to US whites for heart disease (RR=0.82), the second leading cause of death. Among leading causes of death for AN/AI people, the greatest disparities in mortality rates with US whites were observed in unintentional injuries (RR=2.45) and suicide (RR=3.53). All-cause AN/AI mortality has declined 16% since 1980–1983, compared to a 21% decline over a similar period among US whites.

**Conclusion:**

Mortality rates and trends are essential to understanding the health of a population and guiding policy decisions. The overall AN/AI mortality rate is higher than that of US whites, although encouraging declines in mortality have occurred for many cause specific deaths, as well as for the overall rate. The second leading cause of AN/AI mortality, heart disease, remains lower than that of US whites.

Efforts to identify, understand, control and prevent disease and injury have long relied on accurate data on the numbers and causes of death. Collecting, analyzing and tracking mortality of a population is an important function of governments worldwide that allows them to better understand and address the health needs of their populations. In 1954, the Parran report (commissioned by the US Department of the Interior) was the first comprehensive examination of health status—including mortality data—of Alaska Native people completed in the United States (1). Among the report's findings was that infectious disease was the leading cause of death (45.8% of all deaths, many of them from tuberculosis), that the infant mortality rate was greater than 100 deaths per 1,000 births and that life expectancy in 1950 was just 46 years. In light of this report, the Indian Health Service was tasked with establishing adequate health care for all Alaska Native people. Examining causes of death among Alaska Native people during the 1980s, the State of Alaska Section of Epidemiology determined that infectious disease accounted for just 1.3% of deaths, and that injuries were the leading cause of death at 30% (2). More recently, examinations of Alaska Native mortality, through the 1990s and into the early 2000s, has documented that although overall mortality rates continue to decline, deaths from chronic diseases such as cancers are rising, that suicides and homicides remain high and that unintentional injuries and alcohol abuse continue to account for a large proportion of deaths (3,4).

Alaska Native (AN) is used to describe persons whose ancestors historically occupied the area, that is, now the state of Alaska. In 2010, the US Census Bureau estimated there were 138,312 persons living in Alaska who selected the American Indian/AN category alone (or in combination with other racial categories) to classify themselves (5). Although cultural and language differences exist among different AN/AI ethnic groups living in Alaska, the groups are similar in socioeconomic indicators such as income, education, marital status and access to health care. For this report, we examine recent (2004–2008) mortality patterns among these populations, and the changes in mortality rates that have occurred since 1980. In addition, to examine disparities in mortality by race, we compare the AN/AI age-adjusted mortality rates with those of US whites (age-adjusted).

## Methods

Mortality data for AN/AI people who were residents of and died in Alaska were provided by the State of Alaska Bureau of Vital Statistics. Deaths of AN/AI persons were identified in this database by having race designated as Alaska Native (80%), Eskimo (11%), Indian (4%), Canadian Eskimo and Indian (3%), Aleut (2%) and Native Mixed (0.1%). Five-year bridged population estimates (age and sex specific) were used to calculate mortality rates for AN/AI persons living in Alaska. Population estimates for AN/AI persons for the years 1990–2008 utilize the National Center for Health Statistics’ postcensal series by year, age, sex, bridged race and Hispanic origin. Population estimates for 1980–1989 are based on National Cancer Institute's (NCI's) Surveillance Epidemiology and End Results (SEER) program bridged estimates, with age-sex estimates calculated by applying the 1980 census age-sex distribution for AN/AI persons to the years 1980–1984. An average of the 1980 and 1990 census age-sex distributions was applied to 1985–1989 estimates. Data on US whites for rate numerators and denominators came from the NCI's SEER program, available for the years 1969–2006.

All mortality rates were age-adjusted to the US 2000 standard population by the direct method. Mortality rates presented are per 100,000 population. Rate ratios were calculated to compare AN/AI rates to US white rates, with confidence intervals (CIs) calculated around these ratios. Rates were considered significantly different if the 95% confidence interval of the ratio did not contain one, an approximation of a significance test with alpha set to 0.05. All rates reported and compared are age-adjusted.

Mortality rates for the ten leading causes of death were calculated for AN/AI people from among a list of 50 leading causes of death as defined by the National Center for Health Statistics (NCHS), with the addition of alcohol abuse (International Classification of Diseases-10 [ICD-10] code F10). Rates or total deaths were not reported for categorizations yielding fewer than five deaths.

Changes in mortality rates over the period 1980 to 2008 were analyzed. Mantel-Haenszel Chi-square tests for trend, stratified by age group, were used to test for a significant trend over time for each age-adjusted time period (6). Percentage change over time was calculated by subtracting the rate for the first time period from the rate for the most recent time period and dividing by the rate for the first time period.

## Results

### Overall mortality

A total of 3,789 deaths of AN/AI persons living in Alaska were recorded during the period 2004–2008. The ten leading causes of death among AN/AI persons during this period represent about 75% of all AN/AI deaths and are presented in Table I, along with mortality rates and their ratios to US white mortality rates. The AN/AI leading causes are similar to those of the US white top ten, although alcohol abuse, chronic liver disease, and homicide are replaced by Alzheimer's disease, nephritis, and diabetes among US whites for this period, and the ordering of the other seven causes differs slightly. The top three leading causes of death among AN/AI persons accounted for about 48% (1,805) of all AN/AI deaths. Rates for two of the three leading causes (heart disease and injury) are substantially lower in the most recent five-year period (2004–2008) than they were in the earliest period of data (1980–1983).

**Table I:** Leading causes of death, Alaska Native people (2004–2008) versus US whites (2004–2006)

Rank	Cause	ICD-10 definition	Deaths	Proportion (%)	Rate	USW deaths	Proportion (%)	Rate	RR	95% CI
1	Cancer	C00-C97	772	20.4	226.3	1,442,824	23.12	182.4	1.24*	1.15–1.33
2	Heart disease	I00–I09, I11, I13, I20–I51	540	14.3	169.0	1,676,390	26.9	205.1	0.82*	0.75–0.90
3	Unintentional injury	V01–X59, Y85–Y86	493	13.0	97.7	299,968	4.8	39.9	2.45*	2.23–2.69
4	Suicide	X60–X84, Y87.0	252	6.7	42.4	88,895	1.4	12.0	3.53*	3.1–4.00
5	Alcohol abuse	F10	180	4.8	38.7	18,536	0.3	2.4	16.13*	13.83–18.7
6	COPD	J40–J47	172	4.5	57.3	348,784	5.6	43.7	1.31*	1.12–1.53
7	Cerebrovascular diseases	I60–I69	164	4.3	56.7	365,596	5.9	44.5	1.27*	1.09–1.49
8	Chronic liver disease	K70, K73–K74	82	2.2	18.0	71,749	1.2	9.2	1.96*	1.57–2.44
9	Pneumonia influenza	J10–J18	77	2.0	25.5	157,366	2.5	19.0	1.34*	1.06–1.70
10	Homicide	U01–U02, X85–Y09, Y87.1	58	1.5	11.2	27,053	0.4	3.8	2.95*	2.27–3.88
	All others		999	26.4	290.3	1,734,457	27.8	214.6	1.35*	1.53–1.65
	All causes		3,789	100.00	1033.3	6,231,636	100.0	776.6	1.33*	1.29–1.38
Males										
1	Cancer	C00–C97	392	18.9	258.3	748,239	24.5	222.4	1.16*	1.04–1.29
2	Unintentional injury	V01–X59, Y85–Y86	359	17.3	143.7	192,553	6.3	55.0	2.61*	2.33–2.93
3	Heart disease	I00–I09, I11, I13, I20–I51	311	15.0	219	830,291	27.2	256.5	0.85*	0.76–0.97
4	Suicide	X60–X84, Y87.0	189	9.1	62.8	70,306	2.3	19.6	3.20*	2.76–3.72
5	Alcohol abuse	F10	93	4.5	42.4	14,469	0.5	3.9	10.87*	8.80–13.55
6	COPD	J40–J47	80	3.9	59.5	164,571	5.4	51.3	1.16	0.92–1.47
7	Cerebrovascular diseases	I60–I69	72	3.5	58.9	141,685	4.6	44.9	1.31*	1.02–1.69
8	Homicide	U01–U02, X85–Y09, Y87.1	38	1.8	14.5	20,035	0.7	5.5	2.64*	1.87–3.65
9	Pneumonia influenza	J10–J18	37	1.8	33.1	70,079	2.3	22.6	1.46*	1.02–2.08
10	Chronic liver disease	K70, K73–K74	29	1.4	13.2	46,747	1.5	12.7	1.04	0.72–1.51
	All others		471	22.7	306.9	685,398	22.4	234.1	1.31*	1.61–1.77
	All causes		2,071	100.0	1212	3,057,220	100.0	928.5	1.31*	1.24–1.37
Females										
1	Cancer	C00–C97	379	22.01	203.7	694,585	21.9	155.0	1.31*	1.19–1.46
2	Heart disease	I00–I09, I11, I13, I20–I51	229	13.3	129.6	846,099	26.7	164.9	0.79*	0.69–0.90
3	Unintentional injury	V01–X59, Y85–Y86	134	7.8	53.0	107,415	3.4	25.8	2.05*	1.75–2.46
4	Cerebrovascular diseases	I60–I69	92	5.46	54.9	223,911	7.1	43.6	1.26*	1.02–1.55
5	COPD	J40–J47	92	5.4	55.5	184,213	5.9	39.0	1.42*	1.15–1.75
6	Alcohol abuse	F10	87	5.1	36.4	4,067	0.1	1.0	36.10*	28.35–43.6
7	Suicide	X60–X84, Y87.0	63	3.7	21.4	18,589	0.6	5.0	4.28*	3.33–5.55
8	Chronic liver disease	K70, K73–K74	53	3.1	22.6	25,002	0.8	6.0	3.77*	2.87–4.96
9	Pneumonia influenza	J10–J18	40	2.3	22.0	87,287	2.8	16.7	1.32	0.95–1.82
10	Alzheimer's disease	G30	30	1.8	19.8	138,117	4.4	25.1	0.79	0.55–1.13
	All others		518	30.2	267.3	892,570	28.1	171.6	1.56*	1.42–1.70
	All causes		1,717	100.0	886.2	3,174,416	100.0	656	1.35*	1.29–1.42

Note: individual proportions may not sum to 100% due to rounding.

Annual age-adjusted mortality from all causes for AN/AI persons during the period 2004–2008 was 1033/100,000, higher than the rate for US whites (RR=1.33, 95% CI 1.29–1.38). Mortality rates were higher among AN/AI males than AN/AI females (1212 vs. 886), a pattern also observed among US whites. AN/AI mortality rates were significantly higher than those of US whites for nine of the ten leading causes of death. Mortality rates were significantly lower among AN/AI people compared to US whites for heart disease (RR=0.82), as well as for diabetes, the eleventh leading cause of AN/AI mortality and the seventh leading cause of US white mortality (RR=0.69).

### Sex-specific mortality

Rates by sex are also shown in Table II. Sex-specific mortality rates were significantly higher in the AN/AI population than among US whites for both sexes separately for six of the ten leading causes. The overall annual age-adjusted mortality rate among AN/AI males during 2004–2008 was higher than that of females: 1212 versus 826, yielding a rate ratio of 1.47, similar to the rate ratio observed between US white males and females. Cause-specific comparisons of AN/AI mortality to US white mortality were similar to those observed for both sexes combined, with the exception of chronic liver disease, where AN/AI female mortality is quite elevated (RR for females is 3.7, males 1.0), and homicide, which is a leading cause of death among AN/AI males, but not among AN/AI females. Among AN/AI persons, males had higher mortality rates than did females for all leading causes, again excepting chronic liver disease and Alzheimer's disease (not a leading cause of male deaths).

**Table II:** AN/AI deaths by age, sex: 2004–2008

	Male		Female			95% CI	
				
Age	Deaths	Rate	Deaths	Rate	Rate ratio	Lower	Upper
0–4 years							
Unintentional injury	23	80.6	12	44.1	1.8*	1.21	5.29
Perinatal conditions	20	70.1	13	47.8	1.5	0.69	3.21
Congenital abnormalities	12	42	16	58.8	0.7	0.31	1.61
Pneumonia and influenza	–	–	5	18.4	–	–	–
All causes	98	343.3	75	275.6	1.2*	1.27	2.37
Population	28,549	27,214					
5–14 years							
Unintentional	26	48.4	9	18.5	2.6*	1.72	7.88
Suicide	–	–	5	9.5	–	–	–
All causes	35	64.8	21	42.2	1.5*	1.26	3.72
Population	53,284	49,198					
15–24 years							
Suicide	80	145.3	26	50.2	2.9*	2.59	6.29
Unintentional injury	67	122.7	16	29.9	4.1*	3.29	9.81
Homicide	9	15.9	–	–	–	–	–
All causes	181	329.8	63	121	2.7*	2.85	5.06
Population	55,309	51,943					
25–44 years							
Unintentional injury	126	166.8	46	63.2	2.6*	2.93	5.78
Suicide	76	96.2	26	35.3	2.7*	2.69	6.62
Alcohol abuse	29	40.1	39	54.2	0.7	0.46	1.2
Cancer	21	29.4	26	36.5	0.8	0.7	2.23
Heart disease	24	32.9	16	21.4	1.5*	1.26	4.51
Homicide	17	23.7	10	13	1.8	0.83	4.01
Chronic liver disease	6	8.5	19	26.9	0.3	0.24	1.41
HIV	5	6.9	5	7.5	0.9	0.37	3.22
Diabetes mellitus	5	6.9	–	–	–	–	–
Cerebrovascular diseases	–	–	7	9.9	–	–	–
All causes	351	469.1	244	338.7	1.4*	1.83	2.54
Population	75,752	74,260					
45–54 years							
Cancer	61	184.4	45	128	1.4*	1.3	2.82
Unintentional injury	57	171.4	27	75.7	2.3*	1.91	4.78
Heart disease	46	139.8	26	74	1.9*	1.59	4.15
Alcohol abuse	28	84	28	79.3	1.1	0.61	1.86
Chronic liver disease	7	21	17	47.8	0.4	0.24	1.41
Cerebrovascular diseases	5	15.4	8	22.7	0.7	0.29	2.75
COPD	7	21.3	5	14.5	1.5	0.62	6.16
Suicide	22	66	–	–	–	–	–
HIV	5	15.1	–	–	–	–	–
Diabetes mellitus	–	–	6	17	–	–	–
Septicaemia	–	–	5	14.3	–	–	–
All causes	292	881.1	215	610.8	1.4*	1.61	2.29
Population	33,320	35,322					
55–64 years							
Cancer	75	391.4	89	431	0.9	0.88	1.64
Heart disease	52	267.8	25	121.4	2.2*	1.85	4.81
Unintentional injury	23	116.2	6	30	3.9*	2.09	12.65
COPD	14	77.2	12	58.5	1.3	0.8	3.78
Chronic liver disease	13	67.4	12	56.9	1.2	0.72	3.45
Cerebrovascular diseases	11	58.6	6	29.2	2	0.98	7.19
Pneumonia/Influenza	5	27.3	5	26.3	1	0.39	4.72
Diabetes mellitus	–	–	7	33.7	–	–	–
Nephritis	–	–	5	25.5	–	–	–
Septicaemia	–	–	5	23.2	–	–	–
All causes	278	1444.4	225	1099.1	1.3*	1.46	2.08
Population	19,475	20,832					
65–74 years							
Cancer	127	1251.8	107	918.1	1.4*	1.32	2.22
Heart disease	79	782.1	47	403.5	1.9*	1.66	3.41
COPD	29	290.9	28	240.8	1.2	0.89	2.53
Cerebrovascular diseases	15	148.7	14	121.2	1.2	0.73	3.15
Unintentional injury	15	146.5	5	42.6	3.4*	1.59	12.06
Diabetes mellitus	7	68.9	6	51.8	1.3	0.56	4.94
Nephritis	5	47.4	–	–	–	–	–
Pneumonia/Influenza	5	49.6	–	–	–	–	–
All causes	351	3460.1	265	2273.5	1.5*	1.63	2.25
Population	10,490	11,685					
75+ years							
Heart disease	108	1903.2	111	1240.6	1.5*	1.14	1.95
Cancer	106	1779.4	110	1272.3	1.4*	1.07	1.83
Cerebrovascular diseases	38	676.7	56	631.4	1.1	0.7	1.63
COPD	29	502.2	47	538.6	0.9	0.58	1.49
Unintentional injury	22	354	13	143.4	2.5*	1.23	4.94
Pneumonia/Influenza	21	416.1	21	235.3	1.8	0.96	3.27
Parkinson's disease	10	177.3	5	56.4	3.1*	1.06	9.33
Hypertension	8	165.8	7	75.7	2.2	0.78	6.14
Alzheimer's disease	7	144.6	28	311	0.5	0.2	1.08
Diabetes mellitus	7	125.1	10	116	1.1	0.4	2.89
Nephritis	5	93.4	10	113.7	0.8	0.27	2.47
Septicaemia	5	86.3	10	116.1	0.7	0.25	2.22
All causes	485	8619.4	609	6873.8	1.3*	1.11	1.42
Population	6,083	8,727					

Results for categories with fewer than five deaths not presented. *95% Confidence Interval of RR does not contain the value one.

**Table III:** Current top ten causes over time for AN/AI persons, rates per 100,000

Period	Cancer	Heart disease	Unintentional injury	Suicide	Alcohol abuse	COPD	Cerebrovascular disease	Chronic liver disease	Pneumonia/influenza	Homicide	All causes
1980–1983	224.3	270.9	184.1	41.8	28.8	26.2	64.5	28.1	52.2	36.8	1234
1984–1988	243.3	288.1	174.0	49.5	23.2	44.5	65.1	20.4	60.5	22.6	1226.5
1989–1993	245.4	271.9	133.1	46.8	20.6	58.4	67.5	26.8	56.2	18.7	1202.1
1994–1998	248.9	252.0	111.7	42.8	31.7	68.3	84.1	18.3	39.6	16.1	1151.6
1999–2003	239.5	211.6	107.4	34.7	40.9	63.0	66.5	19.4	35.8	16.8	1111.6
2004–2008	226.3	169.0	97.7	42.3	38.7	57.3	56.7	18.0	25.5	11.2	1033.3
% change	NS	−38	−47	NS	34	119	NS	−36	−51	−70	−16
US whites											
1980–1983	203.9	395.1	42.6	13.1	2.0	30.5	85.4	12.9	29.2	6.2	982.7
1984–1988	207.6	362.5	39.1	13.5	1.9	35.8	72.0	11.3	34.2	5.3	955.8
1989–1993	209.9	310.2	35.5	13.0	2.2	39.2	61.5	10.3	34.6	5.6	898.6
1994–1998	202.9	280.2	35.1	12.4	2.3	42.2	59.6	9.6	33.2	4.7	867.5
1999–2003	193.5	243.6	36.4	11.6	2.2	45.8	55.6	9.6	22.5	4.0	834.6
2004–2006	182.4	205.1	39.9	12.0	2.4	43.7	44.5	9.2	19.0	3.8	776.6
% change	−11	−48	−6	−8	20	43	−48	−29	−35	−39	−21

% change=(2004–2008 rate – 1980–1983 rate)/(1980–1983 rate).

### Age-specific mortality

Compared to US whites, AN/AI people had significantly higher rates of all-cause mortality for all age groups under age 75, for each sex separately and for both sexes combined. The greatest disparity between AN/AI people and US whites occurred in the 5–14 age group, largely due to comparatively high AN/AI rates of suicide (RR=13.5) and unintentional injury (RR=5.9). Patterns of disparities among females alone were somewhat different. Females age 25–44 experienced the greatest disparity (RR=3.5) in part due to the comparatively high rate of chronic liver disease among AN/AI females in this age group. AN/AI males had significantly greater all-cause mortality than did females in all age groups.

### Changes over time

Changes in mortality rates over time are presented in Table III. AN/AI all-cause mortality declined 16% during 1980–1983, and 2004–2008 (Table II). This decline was similar to the relative decline (21%) observed for US whites during these same periods. Although a decline in all-cause mortality has occurred in every five-year period examined, the proportional drop was greatest between the most recent two periods (1999–2003 and 2004–2008). However, declines in all-cause mortality mask variations in disease-specific mortalities. Rates of diabetes mellitus mortality among AN/AI people (now the eleventh leading cause) increased 185% between 1980–1983 and between 2004–2008, and chronic obstructive pulmonary disease (COPD) increased 119% during this period. Increases, albeit more modest, were also observed in US white mortality in these categories.

### Death by cause

#### Cancer

For the period 2004–2008, cancer was the leading cause of death among AN/AI people, for both sexes combined as well as for men and women separately, with a mortality rate 24% higher than the corresponding US white rate. Although cancer was not the leading cause of death for every specific age group of AN/AI persons, it was for the sizeable portion of the population aged 45 to 74. Cancer accounted for 20.4% of all AN/AI deaths (n=772). Age specific cancer mortality rate ratios for AN/AI persons compared to US whites were significantly greater for most age groups, and AN/AI men were significantly more likely than AN/AI women to die from cancer in most age groups.

Half the cancer mortality among AN/AI persons was attributable to just three types of cancer: lung and bronchus (31%), colorectal (12%), and pancreatic (7%). Among women, breast cancer was the second leading cause and was responsible for 14% of cancer deaths. This was more than colorectal cancer (12%).

Although mortality from cancer declined significantly among US whites between 1980–1983 and between 2004–2006, the cancer mortality rate among AN/AI persons remained constant between 1980–1983 and between 2004–2008 (224.3 vs. 227.0).

#### Heart disease

Heart disease ranked as the second leading cause of death among AN/AI people and accounted for 14.3% (n=540) of all AN/AI deaths during the period 2004–2008. However, heart disease mortality rates were significantly lower than US white rates. Age-specific heart disease mortality rates among AN/AI were lower than those of US whites for persons 75 and older (RR=0.7) but equivalent or higher for all younger age groups.

Among AN/AI people, heart disease death was nearly twice as common among males as females (RR=1.7), similar to the relation between US white males and females. Heart disease mortality declined 38% between the periods 1980–1983 and 2004–2008, less than the 48% decline that occurred among US whites over a similar interval. Most of the AN/AI decline in heart disease mortality has occurred in recent time periods (1999–2008).

#### Unintentional injury

Unintentional injury was the third leading cause of death for AN/AI people, accounting for 493 deaths (13% of all AN deaths) and an even greater difference from US white rates than observed in the first and second leading causes of death (RR=2.5). However, the disparity was somewhat less in the current period than had been observed during the prior five-year period and substantially less than the disparity observed in 1980–1983, when the unintentional injury mortality rate was more than four times as high among AN/AI people. Overall, AN/AI rates of unintentional injury declined 47% between 1980–1983 and the more recent period of 2004–2008. Significantly higher age-specific rate ratios were found at all ages for AN/AI males, and for the 0–14 and 25–54 age groups for AN/AI females, when compared to US whites. Within AN/AI persons, unintentional injury mortality was higher for males than females for all age groups. The relatively high incidence of unintentional injury mortality among younger AN/AI persons results in unintentional injury being the leading cause of years of potential life lost. Among AN/AI persons between 2004 and 2008, this amounted to 25% (30% among AN/AI males and 17% among AN/AI females) of all years of life lost before age 75.

The top five types of unintentional injury deaths among AN/AI persons (both sexes combined) were poisoning (23% of unintentional injury deaths)—over half of which were overdoses of alcohol or drugs, drowning and watercraft injuries (22%), motor vehicle traffic injuries (11%), exposure to the elements (11%) and ATV injuries (10%).

#### Suicide

Suicide was the fourth leading cause of death among AN/AI people (fourth among males and seventh among females), accounting for 6.7% (n=252, 9.1% of males, 3.7% of females) of deaths during the period 2004–2008 and resulting in a rate 3.5 times that of US whites (42.3 vs. 12.0). Among age groups encompassing 15 to 44 years, AN/AI female suicide rates were 5.3 to 25.6 times those of US white females. Rate ratios comparing AN/AI males to US white males for age groups 15 to 54 years ranged from 2.3 to 8.4. Only for persons over 54 were AN/AI suicide rates comparable to US white rates.

Within the AN/AI population aged 15–44, suicides were about 2.8 times more common among males than among females. Firearms were responsible for 61% of the AN/AI suicides. No changes were apparent in AN/AI suicide mortality rates between 1980–1983 and between 2004–2008.

#### Alcohol abuse

Mental and behavioral disorders due to alcohol abuse are the fifth leading cause of death among AN/AI people, with a mortality rate 16.1 times that of US whites, resulting in 180 deaths (4.8% of all AN/AI deaths). Mortality rates were not significantly higher among AN/AI men than AN/AI women (including for age-specific rate comparisons) in contrast to what has been observed among US whites, where male mortality rates from alcohol abuse are much higher than female rates. Between 1980–1983 and between 2004–2008, AN/AI alcohol abuse mortality rates rose 34%, while US white mortality rates increased 20%.

#### Chronic obstructive pulmonary disease

Chronic obstructive pulmonary disease (COPD) was the sixth leading cause of death in the AN/AI population. The 172 deaths during 2004–2008 resulted in a mortality rate of 57.3, or 1.3 times that of US whites. Although the mortality rate for AN/AI men (59.5) was not significantly higher than that of US white men, the AN/AI female mortality rate of 55.5 was significantly different at 1.4 times the rate of US white females. COPD mortality rates among AN/AI more than doubled (119%) between 1980–1983 and between 1994–1998. There is evidence of possible modest declines in rates since 1994–1998. Among US whites, COPD mortality rates increased 43% between 1980–1983 and between 2004–2006.

#### Cerebrovascular disease

Cerebrovascular disease was the seventh leading cause of death among AN/AI people and accounted for 4.3% (n=164) of all AN/AI deaths during 2004–2008. The AN/AI mortality rate of 56.7 was significantly higher than that observed among US whites (44.5; RR=1.27). Rates by age group revealed that only in the 25–44 age group was the overall rate significantly higher among AN/AI persons than among US whites. No significant differences by sex were observed among AN/AI people. Although cerebrovascular disease mortality rates declined 48% between 1980–1983 and between 2004–2006 among US whites, no significant change or clear trend among AN/AI rates occurred during that time.

#### Chronic liver disease

Chronic liver disease and cirrhosis was the eighth leading cause of death overall among AN/AI people accounting for 2.2% (82) of deaths during 2004–2008, yielding a mortality rate 2.0 times that of US whites. This difference was a consequence of the comparatively higher rate among AN/AI women, whose mortality rate was a significant 3.8 times the rate of US white women.

Differences in chronic liver disease mortality were most evident in women, age 25 to 64 years, where AN/AI mortality risks were 4.3 to 11.0 times those of US whites. Female mortality rates were higher than male mortality rates for AN/AI persons of all age groups with deaths, although the difference was significant (RR=3.2) only for persons age 25–44. The AN/AI chronic liver disease mortality rate declined 36% between 1980–1983 and between 2004–2008. US white mortality declined 29% over a similar period.

#### Pneumonia/influenza

When mortality data was first systematically collected in the 1950s, pneumonia/influenza was among the leading causes of death and a large contributor to years of potential life lost. In the most recent period of 2004–2008, it was the ninth leading cause of death and mostly occurred among the elderly. Pneumonia/influenza was responsible for 2% (n=77) of all deaths during this period, resulting in an age-adjusted mortality rate 1.3 times that of US whites.

The AN/AI mortality rate for pneumonia/influenza declined 51% between 1980–1983 and between 2004–2008.

#### Homicide and legal intervention

Homicide and legal intervention was the tenth leading cause of death among AN/AI people (2.9 times the rate of US whites), ranking eighth among males and twelfth among females. Homicide deaths are a result of injuries inflicted by another person with intent to harm or kill. Deaths from legal intervention include injuries inflicted by the police or other law enforcement agents, including military on duty, in the course of arresting or attempting to arrest lawbreakers, suppressing disturbances, maintaining order and in other legal actions. For 2004–2008, 98% of the deaths (n=58) in this category were due to homicides (7). Thirty-eight percent of the deaths involved firearms.

Between the periods of 1980–1983 and of 2004–2008, mortality rate due to homicides and legal interventions dropped 70% among the AN/AI population, compared to a 39% decline experienced by US whites during a similar period.

## Discussion

All-cause mortality among AN/AI people declined 16% between 1980–1983 and between 2004–2008. Among the ten leading causes of death, mortality declined in five (often dramatically), remained unchanged in three and increased in two.

Although all-cause mortality rates among AN/AI people declined during 2004–2008, disparities within the US white population persist. Overall, AN/AI age-adjusted mortality remains about 33% higher (a relative difference that has varied little since 1980–1983); and nine of the ten leading causes of AN/AI death (accounting for about 60% of AN/AI deaths) are significantly greater than the corresponding mortality rates of US whites. The mortality rate of heart disease, the second leading cause of death among AN/AI people, remains lower than the US white rate, as has been observed in previous reports. Rates for both populations have declined since 1980–1983, although the decline among AN/AI people has been at a slower pace than has the decline among US whites. Heart disease is the leading cause of death among persons age 75 and older in both populations. Because of the much lower AN/AI rate (compared to US whites) in this age group (RR=0.7), the *overall* age-adjusted AN/AI heart disease mortality rate is lower than that of US whites. However, in all younger age groups, AN/AI mortality due to heart disease is as high or higher than US white rates, and the combined younger age groups of AN/AI people suffer more heart disease deaths than does the oldest age group. This pattern suggests that rates of heart disease among younger AN/AI persons may be higher than rates experienced previously by older generations, and deaths due to heart disease may thus be likely to increase (relative to US whites) in coming years as these younger AN/AI persons age.

COPD mortality has increased more since 1980–1983 than any other top ten leading cause of death and with greater increases than those observed among US whites. However, most of the rate increases occurred in the 1980s and early 1990s; recent modest but steady declines in COPD mortality may indicate a reversal of that trend.

Cancer remains the leading cause of AN/AI death and, unlike US whites (who have experienced a small decline in mortality over the last 30 years), there is little evidence that cancer mortality is diminishing among AN/AI people. As cancer consists of many diseases, some types of cancer deaths have experienced encouraging declines. Many cancers take years to develop, and lifestyle changes occurring in recent years may not become evident in reduced cancer mortality for some time. For example, smoking prevalence among Alaska Native people has been declining since at least 1991, but neither lung cancer incidence nor mortality has yet to decline (8).

For AN/AI persons under age 70, the leading cause of death was injuries (unintentional and suicide/homicide), with rates much greater than US white rates. Suicide rates among persons aged 15–24 were 9.3 times as high among AN/AI persons as US whites, and 4.3 times as high among AN/AI adults aged 25–44. Homicide mortality rates among AN/AI adults in these age groups were also elevated, at 1.8 and 3.3 times the rates of US whites; unintentional injury mortality rates were 1.9 and 3.0 times those of US whites for these age groups.

AN/AI persons’ disproportionate exposure to the Alaskan natural environment contribute, in part, to the observed elevated unintentional injury mortality rates. About 67% of AN/AI persons live in rural areas of the state (vs. 37% of the Alaska white population) where work, travel and subsistence food gathering place them at increased risk of any death. More than 40% of unintentional injury deaths arise from causes directly associated with Alaska's extreme natural environment. Poisoning deaths (over half of which are alcohol or drug overdoses) account for about 20% of unintentional injury deaths.

Encouragingly, unintentional injury and homicide rates have declined significantly since 1980–1983. Unfortunately, suicide rates have remained relatively constant and disturbingly high.

## Limitations

Mortality rates are only reported for categories in which at least five deaths occurred. For some age-sex-disease classifications with very small numbers of deaths, a change of a single death could have a large effect on reported mortality rates.

There is often concern about the under-reporting of minority groups on death certificate data; however, a 1996 study estimates that under-reporting of Alaska Native people deaths on death certificates is low (5%) (9). In previous examinations of AN/AI mortality, we have also compared AN/AI mortality to non-Native Alaskans. However, these earlier comparisons indicate that mortality rates and patterns among non-Native Alaskans closely resemble those of US whites, and we chose to not present non-Native Alaskan data in this report.

For convenience and ease of understanding, we have most often presented changes in mortality by comparing the most recent five-year period (2004–2008) to an earlier period (1980–1983). Changes in the mortality rates examined have not, necessarily, steadily increased or decreased over that near-30-year span. Although this manuscript has focused, in large part, on the 10 leading causes of death, fully 30% of AN/AI deaths are from other causes—more than the proportion of deaths attributed to any single leading cause of death.

## Conclusion

Although tremendous gains have been achieved in many of the leading causes of AN/AI mortality, higher rates persist for overall mortality and for nine of the ten leading causes of AN/AI death in comparison to US whites. Large mortality rate differences remain in unintentional and intentional injuries despite continued encouraging declines in the AN/AI rate. In part, these differences represent the inherently higher risks presented by Alaska's harsh natural environment interacting with a large proportion of AN/AI people's rural lifestyle. Suicide also remains disturbingly high, with the current mortality rate similar to that of the early 1980s. Increases in AN/AI COPD mortality rates observed in prior periods, however, appear to have halted and may be beginning to decline. Cancer remains the leading single cause of death, responsible for 20% of AN/AI deaths. Monitoring mortality rates and their trends remains essential for understanding the health status of a population, identifying potential areas for preventive programs and policies, and for gaining insight into the efficacy of policy changes or public health campaigns.
